# Effect of confinement pressure on bearing capacity of two samples of square and strip footing (numerical study)

**DOI:** 10.1186/2193-1801-3-593

**Published:** 2014-10-09

**Authors:** Aarash Hosseini

**Affiliations:** Islamic Azad University, Arak branch, Arak, Iran

**Keywords:** Footing bearing capacity, Lateral pressure, Cohesion, Friction angle

## Abstract

This paper presents the results of modeling tests of the Effect of Confinement Pressure on footing bearing capacity of two kinds of square and strip footing. Footings bearing capacity depends upon many factors including soil kind, depth, form and kind of loading. Soil behavior is variable regarding the kind of loading and the kind of deformations in that can have great importance in the amount of bearing capacity. The kind of deformations depends on the amount of pressure on soil in the past and present. Therefore, studying the role of stress way, which is subject to the amount of confinement pressure on soil, will have an important role in identifying soil behavior. In this study, primarily the effect of confinement pressure on the cohesion and friction angle is studied. Then the effect of both on the bearing capacity with the Meyerhof and Terzaghi methods is evaluated. By using Plaxis software, changes of shearing resistance parameters of both samples different Confinement pressures are studied and bearing capacity of two kinds of square and strip footing has been computed and compared. This study indicated that the amount of bearing capacity by increasing lateral pressure increased, and this increasing is more in grain soil than cohesion one.

## 1 Introduction

The practices of improvement of soil by different techniques have been received as of late by Civil Engineering experts. Utilization of sites with marginal soil properties has been expanded due to the need accessibility of good construction sites. Because of this, improve foundation soil bearing capacity has risen noticeably. One method of improving soil capacity is soil confinement. Using metalcell, geocell are the current improvement this field to supply confinement to the soil. Civil Engineering professionals have applied these novel approaches efficiently in several fields of Geotechnical engineering; however they have not obtained much attention in foundation applications. Over the last few decades, via consideration of soil and structure interaction great strides in the modification of existing forms of foundations along with the development of new and unconventional types of foundation systems have occurred. This results in a system utilizing the form and material strength that is more realistic in performance. One of these novel methods is the lateral confinement of cohesion less soil. The effect of lateral confinement on bearing capacity, especially on sandy soil has been studied by many researchers.

Confining the soil is reductions in the settlement have been concluded by these researchers, and hence an increase is in the bearing capacity of the soil.

Arrive at optimum dimensions of the cell have been concluded by a series of model plate loading tests on circular footings supported over sand-filled square-shaped paper grid cells to identify different modes of failure (Rea and Mitchell (1978)).

experimental study concerning a method of improving the bearing capacity of the strip footing resting on sand sub-grades utilizing vertical non extensible reinforcement were presented by Mahmoud and Abdrabbo (1989). The test results indicate that the bearing capacity of sub-grades and modifies the load– displacement behavior of the footing is increased with this type of reinforcement.

The laboratory-model test results for the bearing capacity of a strip foundation supported by a sand layer reinforced with layers of geogrid were investigated by Khing et al. (1993).

The ultimate bearing capacity of strip and square foundations supported by sand reinforced with Geogrid were studied by Puri et al. (1993). The ultimate bearing capacity of surface strip foundations on geo grid-reinforced sand and unreinforced sand were presented by Omar et al. (1993a, [b]). the use of vertical reinforcement along with horizontal reinforcement consisted of a series of interlocking cells, constructed from polymer Geogrids, which contain and confine the soil within its pockets were investigated by Dash et al. (2001b). Mandal and Manjunath (1995) used geo grid and bamboo sticks as vertical reinforcement elements, also they studied their effect on the bearing capacity of a strip footing. Rajagopal et al. (1999) have studied the strength of confined sand, the influence of geo cell confinement on the strength and stiffness behavior of granular soils. An experimental study on the bearing capacity of a strip footing supported by a sand bed reinforced with a geo cell mattress was performed by Dash et al. (2001a). Strip foundations but reinforced with different materials such as steel bars also was studied by Several authors (Milovic, 1977; Bassett and Last, 1978; Verma and Char 1986), steel grids (Dawson and Lee, 1988; Abdel-baki et al. 1993), geotextile (Das 1987), and geogrids (Milligan and Love, 1984; Ismail and Raymond, 1995). The results of laboratory model tests on the effect of soil confinement on the behavior of a model footing resting on Ganga sand under eccentric – inclined load were presented by Vinod Kumar Singh et al. Confining cells with different heights and widths have been used to confine the sand.

## 2 Modeling

In this research, Plaxis software has been used for numerical modeling. PLAXIS is a three-dimensional finite element program especially developed for the analysis of foundation structures, including off-shore foundations. It combines simple graphical input procedures, which allow the user to automatically generate complex finite element models, with advanced output facilities and robust calculation procedures. The program is designed such that a user can analyze complex constructions after only a few hours of training. This program can model the soil behavior under loading as well as it happens in the nature.

In order to simulating soil behavior, hardening soil model has been used. Used parameters for samples are presented in Table 1.

**Table 1:** **The specified parameters of soil samples used in numeral analyses**

Sample	C	ϕ	
1	0	30	17.3
2	25	5	16.4
Unit	kN/m^2^	°	kN/m3

The boundary condition is modified in one of vertical sides of the model as grid along × direction and transferable along y direction and beneath the model is grid along y direction and transferable along × direction. So, in addition to preservation of balance of the entire model in horizontal side, it’s move along with vertical will also be released that are the direction of weight power and enforcing load.

The following assumptions have been considerable for simpler analysis.

1. The issue has been analyzed as an axisymmetry model.
2. Considering long term behavior of soil, the sample has been studied in drained condition.
3. The study has been carried out parametrical.

## 3 Methodology and the results of analysis

Several different approaches in determination of the bearing capacity of shallow foundations have been generally employed in the past decades. The famous triple-N formula of them is Terzaghi, and can be written as given in equation (1)
1

Where, *q*_ult_ is the ultimate bearing capacity of soil mass, c is the cohesion, q is the surcharge pressure, B is the foundation width and *γ* is the unit weight of soil mass. Similarly *N*_*c*_, *N*_*q*_, *N*_*γ*_ are bearing capacity factors, which are functions of the soil friction angle. The second and third terms in equation (1) have been known as the main contributor to the bearing capacity of shallow foundations on non-cohesive soils. Different investigators such as Terzaghi (1943), Meyerhof (1963), Hansen (1970), Vesic (1973), Bolton and Lau (1989) suggested values for the third factor.

Although all these methods are generally based on a limit equilibrium solution, there are differences between their assumptions for boundary conditions and consideration of the soil weight effect. taking several assumptions in to account comp uted the third bearing capacity factor i.e. *N*_*γ*_. Terzaghi (1943) assumed that, the components of bearing capacity equation can be safely superposed. Meyerhof (1951, 1963), proposed a bearing-capacity equation similar to that of Terzaghi but included a shape factor s-q with the depth term Nq. He also included depth factors and inclination factors.

Beside these assumptions, almost all conventional methods assume a constant value of soil friction angle to compute the bearing capacity factors. Generally, in calculating the footing bearing capacity, the condition of lateral pressure on soil is not considered. The amount of friction angle and cohesion are counted based on resulted average of some experimental tests while the structural value of foundation is ignored. However, foundation can have major effect on the amount of stress in the soil [Abdel-baki et al. (1993), Das and Omar (1994), Das et al. (1996), De Beer (1970), Fragaszy and Lawton (1984), Meyerhof (1953, 1965)].

In this research, by using Plaxis software, changes of shearing resistance parameters of both samples confinement pressure of 100,300,600 ,1000 ,1500 and 2000 kN/m^2^ are studied and bearing capacity of two kinds of square and strip footing based on Terzaghi and Meyerhof methods has been computed and compared.

These numerals analysis attempt to provide a better understanding of the effect of confinement pressure on the bearing capacity.

In order to study the effect of confinement pressure on bearing capacity and its parameters, at first, the changes of coherency and the friction angle has been studied and represented in Table 2.

**Table 2:** **Amount of coherency and friction angle**

Sample	1	ϕ _in_= 30	2	ϕ _in_= 5
σ _3_	ϕ	C	ϕ	C
100	30.2	8.13	7.86	46.56
300	34.1	0	7.86	46.56
600	31.4	8.16	6.7	60.3
1000	31.1	26.34	5.4	82.65
1500	29	116.6	5	103.1
2000				

**Figure 1:**
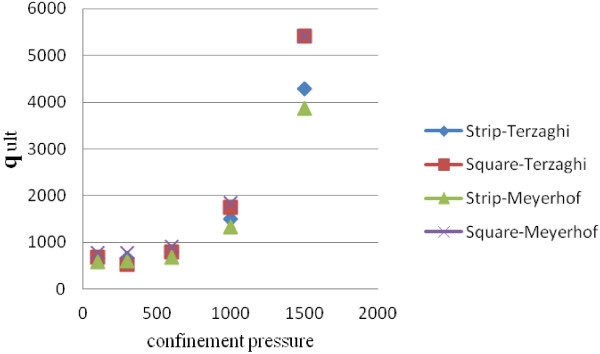
**Bearing capacity changes in sample 1.**

**Figure 2:**
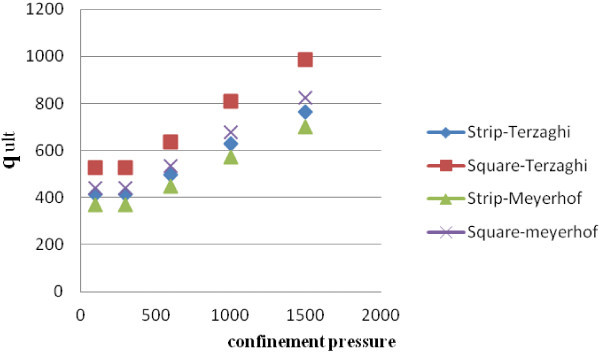
**Bearing capacity changes in sample 2.**

After that, a square footing and a strip one in dimensions of 2 × 2 m and 2 × 10 m has been and the coefficients of bearing capacity and ultimate bearing capacity based on Terzaghi and Meyerhof methods, which have the most applications, by considering the amount of friction angle and coherency obtained in confinement pressure have calculated. The obtained result has been presented in Tables 3and 4 and Figures 1 and 2.

**Table 3:** **The coefficients and the ultimate bearing capacity in sample 1**

Sample	1												
σ _3_	ϕ	C	Terzaghi						Meyerhof				
			a	Nq	Nc	Nγ	q _ult_		Nq	Nc	Nγ	q _ult_	
							Strip	Square				Strip	Square
100	30.2	8.13	3.38	22.89	37.63	20.34	671.99	690.5	18.74	30.50	16.12	585.50	775.21
300	34.12	0	4.03	36.86	52.97	37.19	669.41	535.5	29.72	42.42	31.61	609.39	770.80
600	31.42	8.16	3.56	26.46	41.7	25	790.36	802.4	21.56	33.67	19.83	689.15	919.03
1000	31.12	26.34	3.52	25.53	40.65	24.39	1509.7	1743	20.82	32.85	18.84	1334.12	1852.66
1500	29	116.6	3.20	19.89	34.11	17.1	4285.2	5417	16.37	27.75	13.17	3858.83	5401.99
2000

**Table 4:** **The coefficients and the ultimate bearing capacity in sample 2**

Sample	2												
σ _3_	ϕ	C	Terzaghi						Meyerhof				
			a	Nq	Nc	Nγ	q _ult_		Nq	Nc	Nγ	q _ult_	
							Strip	Square				Strip	Square
100	7.86	46.56	1.37	2.17	8.516	0.894	412.59	528.3	2.03	7.45	0.20	368.59	441.89
300	7.86	46.56	1.37	2.17	8.516	0.894	412.59	528.3	2.03	7.45	0.20	368.59	441.89
600	6.7	60.3	1.31	1.94	8.001	0.757	496.08	638.1	1.82	7.02	0.14	447.38	533.14
1000	5.4	82.65	1.24	1.71	7.47	0.553	627.35	810.6	1.62	6.58	0.08	572.01	677.15
1500	5	103.1	1.22	1.64	7.315	0.494	762.74	987.1	1.56	6.46	0.07	698.31	825.00
2000

In order to Study The effect of confinement pressure on bearing capacity, The ultimate bearing capacity related to each Sample has been presented at to pressure of 100 and 2000 kN/m^2^ in Table 5 and are compared by applying bearing capacity ratio coefficient (BCR).

**Table 5:** **Comparing the bearing capacity of samples**

	Terzaghi						Meyerhof					
	Strip		Square		BCR		Strip		Square		BCR	
	q _ult_		q _ult_		Strip	Square	q _ult_		q _ult_		Strip	Square
σ _3_	2000	100	2000	100			2000	100	2000	100		
Sample												
1	4285	672	5417	690.5	6.38	7.84	3858.83	585.50	5401.99	775.21	6.59	6.97
2	762.7	412.6	987.1	528.3	1.85	1.87	698.31	368.59	825.00	441.89	1.89	1.87

## 4 Conclusion

Based on results obtained, it is observed that bearing capacity in sample 1 in strip footing with Terzaghi method increases 6.59 fold this increase in square footing is with Terzaghi and Meyerhof method 7.84 and 6.79 fold, respectively. Also in sample2 in strip footing with Terzaghi method increases 1.85 fold and with Meyerhof method increases 1.89 fold this increases in square footing is 1.87 Fold with Terzaghi and Meyerhof methods. By comparing obtained results, it way resulted that increasing confinement pressure in grain soils have more effect on increasing bearing capacity.
